# Combined ICP-OES and XPS analysis to evaluate the [AlO_4_]^0^ concentration in quartz: limiting the formation temperature of quartz

**DOI:** 10.1039/d3ra03701k

**Published:** 2023-08-25

**Authors:** Mengmeng Shen, Mulin Huang, Zhiyun Lu, Xuemei He

**Affiliations:** a School of Gemmology, China University of Geosciences Beijing 100083 China hexuemei3127@126.com; b National Gems and Jewelry Technology Administrative Center Beijing 100013 China; c School of Earth Sciences, Zhejiang University Hangzhou 310027 China

## Abstract

A proposed quartz thermometer is based on the concentration of [AlO_4_]^0^ tetrahedra determined by combining inductively coupled plasma optical emission spectrometry (ICP-OES) with X-ray photoelectron spectroscopy (XPS) data. The concentration of [AlO_4_]^0^ tetrahedra in the quartz lattice (*C*_[AlO_4_]^0^_/ppm) and the formation temperatures of quartz (*T*_Q_/°C) from agate, gold deposits, and granodiorite are calculated by *C*_[AlO_4_]^0^_ = *C*_Al total_ (ppm) × *k* and *T*_Q_ (°C) = 3.6 × *C*_Al total_ (ppm) × *k* + 33.0, respectively. Where *C*_Al total_ is the total Al concentration of quartz measured by ICP-OES and *k* is the relative percentage of [AlO_4_]^0^ tetrahedra in the quartz lattice and can be obtained by fitting the Al(2p) XPS spectrum. The obtained formation temperatures of quartz (*T*_Q_) agree well with the equilibrium formation temperature (*T*_E_) calculated by oxygen isotope data. By comparing the relative positions of the two temperature curves of quartz (*T*_Q_ and *T*_E_), the composition of the mineral-forming fluid can be inferred. The proposed quartz thermometer can be applied to quartz formed under equilibrium conditions and in Al-saturated environments over a wide temperature range (152–566 °C). The use of the quartz thermometer effectively eliminates interference from different fluid compositions and satisfies the requirements of convenience and economy.

## Abstract 2

The formation temperature of quartz (*T*_Q_/°C) are calculated by *T*_Q_ (°C) = 3.6 × *C*_Al total_ (ppm) × *k* + 33.0.

## Graphical abstract

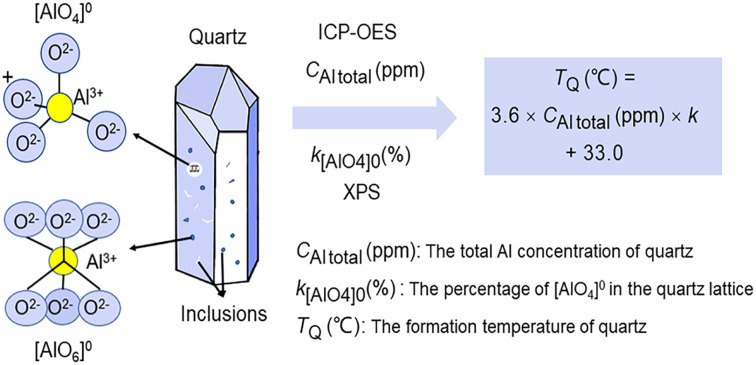

## 1. Introduction

As the second most abundant mineral in the Earth's continental crust, quartz is widely found in sedimentary, metamorphic, and igneous environments.^1–9^ Due to limitations in charge and ionic radius, only a small number of ions can replace Si^4+^ in the quartz lattice.^10^ The [AlO_4_]^0^ center is caused by the substitution of Al^3+^ for Si^4+^ and the formation of O^1−^ by replacing Si^4+^ with an electron hole at one of the four closest O^2−^ ions.^11–20^ The [AlO_4_]^0^ center consists of a hole trapped in a non-bonding orbital of an O ion located adjacent to the substitutional Al.^21^ The precursor state of this center is the diamagnetic [AlO_4_/M^+^]^0^ associated with the adjacent charge-compensated cations M^+^ (H^+^, Li^+^, Na^+^).^21^ During γ-irradiation of quartz at 295 K, the M^+^ ions may diffuse away to produce paramagnetic [AlO_4_]^0^. In the more open spaces in the structure or by micro-inclusions of Al-bearing phase, Al^3+^ exists outside the silica-oxygen skeleton as [AlO_6_]^0^ octahedra (six-coordination), playing a role similar to that of general cations such as Mg^2+^ and Fe^2+^.^22,23^ The formation temperature of quartz can be estimated by various techniques such as oxygen isotope data, micro-thermometry of fluid inclusions, and the titanium-in-quartz geothermometer.^17,24–33^ Perry (1963) observed that the Al content in quartz from contact-metamorphosed quartz-aluminum silicate rocks decreased regularly with increasing distance from the intrusion, suggesting that the Al solubility in quartz may be temperature-dependent.^34^ The result of chemical equilibrium simulations show that factors such as pressure, growth rate, and fluid composition have no significant effect on the Al content in quartz.^35^ The Al content in quartz is mainly controlled by temperature, so the Al-in-quartz thermometer mainly considers temperature.^36^ Dennen *et al.* (1970) found that under Al-saturated and equilibrium crystallization conditions, the Al content in quartz varies with crystallization temperature over a wide range of temperatures (80–900 °C).^35^ The formation temperature (*T*_Q_P__/°C) of both natural and synthetic quartz crystals changed with their total Al concentrations (*C*_Al total_/ppm), as described by the equation of:

1 *T*_Q_P__(°C) = 3.6 × *C*_Al total_ (ppm) + 33.0

The equation above yields a considerably high estimate for the crystallization temperature. This may be because the total Al content (*C*_Al total_/ppm) not only include the [AlO_4_]^0^ tetrahedra in the quartz structure but also [AlO_6_]^0^ octahedra in the more open spaces in the structure or by micro-inclusions of Al-bearing phases.^37–39^ Apparently, it is controversial to calculate the formation temperature of quartz (*T*_Q_P__) directly using the total Al concentration obtained by bulk analytical methods. To effectively avoid the influence of the [AlO_6_]^0^ octahedra, the concentration of [AlO_4_]^0^ tetrahedra in the quartz lattice can be directly quantified through electron paramagnetic resonance (EPR).^40^ However, prior to conducting EPR testing, quartz must undergo a complex heat treatment and irradiation process to convert the diamagnetic [AlO_4_/M^+^]^0^ to the paramagnetic [AlO_4_]^0^, enabling their detection through EPR.

In this study, an alternative method for distinguishing between [AlO_4_]^0^ tetrahedra in the quartz lattice and [AlO_6_]^0^ octahedra in the more open spaces in the structure or by micro-inclusions of Al-bearing phases is the XPS analysis of quartz. Unlike EPR testing, XPS analysis does not require complex pre-processing to exclude the effects of impurity inclusions in quartz. The relative percentage of [AlO_4_]^0^ tetrahedral (*k*) in the quartz lattice is determined by fitting the Al(2p) XPS spectrum.^41–46^ The concentration of [AlO_4_]^0^ tetrahedra (*C*_[AlO_4_]^0^_/ppm) in the quartz lattice is obtained by multiplying the total Al concentration of quartz (*C*_Al total_/ppm) obtained through ICP-OES by the relative percentage of [AlO_4_]^0^ tetrahedra (*k*) from XPS analysis. In this research, fragments containing quartz with varying geological backgrounds and formation temperatures were selected to evaluate the feasibility of the quartz thermometer. The formation temperature of quartz (*T*_Q_) was determined by analyzing the concentration of [AlO_4_]^0^ tetrahedra in the quartz lattice by a combination of ICP-OES and XPS techniques. The calculated results (*T*_Q_) were then compared to the equilibrium formation temperatures (*T*_E_) obtained by analyzing the oxygen isotopic compositions of the same samples. This quartz thermometer is easy to operate, reduces costs, and eliminates interference from the composition of the mineralizing fluid.

## 2. Materials and methods

**Fig. 1:**
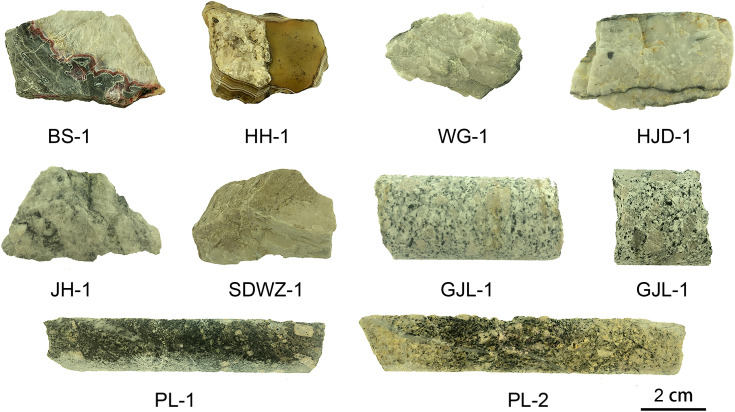
Quartz bearing fragments selected from sediments of hydrothermal agate, gold bearing quartz vein, and granodiorite in China.

The analyzed material includes quartz-bearing fragments from eight occurrences across China, as shown in Fig. 1 and Table 1. The origins of these fragments are epithermal, hydrothermal, and magmatic. Their formation temperatures span low, medium, and high ranges (Table 1). Agate BS-1 and HH-1 originate from primary and secondary agate deposits, respectively, with formation temperatures below 220 °C.^47,48^ The gold-bearing quartz veins are obtained from various types of gold deposits, including orogenic gold, epithermal gold, and porphyry copper polymetallic deposits.^49^ Furthermore, granodiorites containing quartz exhibiting a broad temperature range between 250 and 733 °C have also been selected.^50^ The quartz-bearing fragments were carefully crushed before individual quartz pieces are hand-picked under a binocular microscope. These pieces were then ground into 300 mesh powders. The purity of quartz powder reaches more than 95%. To address the limitation of the micro-area analysis, quartz powders were selected for ICP-OES, XPS, and oxygen isotope tests, respectively.

**Table 1:** Details of the quartz bearing rocks with different geological backgrounds

No.	Description	Genetic type	Occurrence	Temperature (°C)
BS-1	Primary agate	Epithermal	Baoshan, Yunnan	87–152
HH-1	Secondary agate	Epithermal	Xunke, Heilongjiang	203–220
WG-1	Gold bearing quartz vein	Hydrothermal	Wangu gold deposit, Hunan	225–385
HJD-1	Gold bearing quartz vein	Hydrothermal	Huangjindong gold deposit, Hunan	160–397
JH-1	Gold bearing quartz vein	Hydrothermal	Jinhong gold deposit, Hunan	258–397
SDWZ-1	Gold bearing quartz vein	Epithermal	Sandaowanzi gold deposit, Heilongjiang	160–385
PL-1	Gold bearing quartz vein	Hydrothermal	Pulang copper–gold deposit, Yunnan	115–450
PL-2	Gold bearing quartz vein	Hydrothermal	Pulang copper-gold deposit, Yunnan	115–450
GJL-1	Granodiorite	Magmatic	Guojialing, Shandong	250–733
GJL-2	Granodiorite	Magmatic	Guojialing, Shandong	250–733

The total Al content of the quartz powder was detected by ICP-OES (JY Ultima2C). A mixture of 100 mg of quartz powder and 400 mg of anhydrous lithium metaborate was melted in a muffle furnace at 1000 °C for 15 minutes. The cooled silica mixture was added to 50 ml of 5% aqua regia solution at 80 °C. After the silica mixture was completely dissolved and cooled, the volume was adjusted to 100 ml with 5% aqua regia solution. The solution was left for 24 hours, and then analyzed by ICP-OES. The ICP-OES parameters were as follows: forward power of 1200 W, plasma gas flow rate of 14.0 L min^−1^, nebulizer gas flow rate of 1.0 L min^−1^, sample uptake speed of 1.0 ml min^−1^, the integration time of 10–15 s at low and high wavelength ranges. A concentric nebulizer was used with a cyclonic spray chamber. The analytical wavelength for the Al element was 396.152 nm. The measurement was controlled by ICP jy5.1 software. The relative error of the results is less than 3%.

The Al(2p) spectra of the quartz powders were obtained by a Thermo Scientific ESCALAB 250 Xi+ spectrometer. The C(1s) peak (284.6 eV) was used to calibrate the position of the binding energy. The data was processed by using the CaseXPS software, Shirley background, Gaussian : Lorentz function = 60 : 40 to fit the XPS data. The test error is ±0.1%, and the relative error of Al(2p) peak fitting is less than ±1.0%.

The oxygen isotope of quartz powders was analyzed by a 253plus gas isotope ratio mass spectrometer at Nanjing Hongchuang Geological Exploration Technology Service Co., Ltd. The quartz powders were placed on a nickel sample holder and heated with a 20 W CO_2_ laser. BrF_5_ was used as the fluorinating agent. The released oxygen was converted to CO_2_ and then admitted online to the mass spectrometer. The accuracy of the method is ±0.2‰, and the relative error of the temperature calculated by the error transfer formula is ±4.0%.

## 3. Results and discussion

### 3.1 ICP-OES data

The total Al contents of the quartz powders (*C*_Al total_) are determined by ICP-OES and shown in Table 2. Quartz pieces from agate deposits exhibit a low total Al content, ranging from 182–643 ppm. In contrast, the total Al contents of quartz pieces from gold deposits are more variable, ranging from 559 to 5070 ppm. Notably, the total Al content of quartz pieces in granodiorite deposits can be as high as 8660 ppm, and it is significantly greater than that of quartz from other deposits. The total Al concentration of quartz (*C*_Al total_) obtained by ICP-OES represents the total concentration of [AlO_4_]^0^ tetrahedra in the quartz lattice and [AlO_6_]^0^ octahedra in the impurity inclusions. Therefore, direct measurement of the concentration of [AlO_4_]^0^ tetrahedra (*C*_[AlO_4_]^0^_/ppm) in the quartz lattice by ICP-OES is not feasible.

**Table 2:** Oxygen isotope data, Al, and [AlO_4_]^0^ concentration of investigated quartz. Temperatures of quartz formation “*T*_met_”, “*T*_oc_”, “*T*_mag_” and “*T*_Q_” were calculated from oxygen isotope data and the quartz thermometer, respectively

No.	*δ* ^18^O_SMOW_ (‰)	*T*_met_ (°C)	*T*_oc_ (°C)	*T*_mag_ (°C)	*C*_Al total_ (ppm)	*k* (%)	*C*_[AlO_4_]^0^_ (ppm)	*T*_Q_ (°C)
BS-1	17.0	59	132	248	182	18.2	33	152
HH-1	13.8	78	169	332	643	7.3	47	202
WG-1	18.3	52	120	222	559	10.9	61	253
HJD-1	16.9	59	133	250	885	6.7	59	245
JH-1	15.8	66	145	275	1100	6.4	70	285
SDWZ-1	−2.8	291	2286	−395	4770	1.7	81	325
PL-1	12.7	85	184	373	5070	1.8	91	361
PL-2	11.7	92	199	417	4900	2.3	113	440
GJL-1	9.8	107	232	535	8660	1.7	147	562
GJL-2	9.5	110	237	560	8230	1.8	148	566

### 3.2 XPS data

**Fig. 2:**
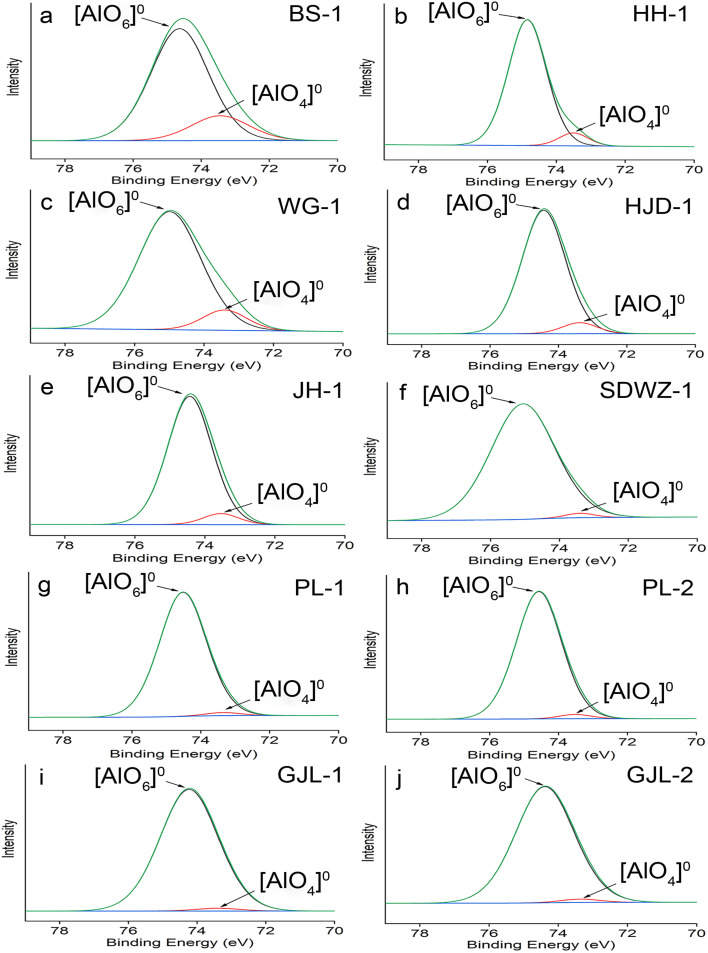
Shirley background-subtracted XPS spectra in the Al region of quartz from agate deposits (a and b), gold deposits (c–h), and granodiorite deposits (i and j). The black and red peaks represent [AlO_4_]^0^ tetrahedra and [AlO_6_]^0^ octahedra, respectively.

The Al(2p) spectra of our specimens were fitted using Gaussian–Lorentzian (GL) = 60 : 40. The results of XPS and peak fitting are given in Fig. 2 and Table 3. In the region of Al(2p), the Al^3+^ binding energy values range from 73.4 to 74.2 eV in tetrahedral coordination and from 74.1 to 74.9 eV in octahedral coordination.^51^ The deconvolution of the Al(2p) spectrum in our work produced two components (73.3–73.5 and 74.2–75.0 eV), which accords well with the results of previous studies, so the binding energies at 73.3–73.5 and 74.2–75.0 eV are caused by the [AlO_4_]^0^ tetrahedra in four-coordination and [AlO_6_]^0^ octahedra in six-coordination, respectively.^11,22,23^

**Table 3:** Semi-quantitative calculation of aluminum with different coordination modes in the quartz. (The results retained to one decimal place.)

No.	Peak	Position (eV)	FWHM (eV)	% area	No.	Peak	Position (eV)	FWHM (eV)	% area
BS-1	1	73.4	2.0	18.2	SDWZ-1	1	73.4	1.0	1.7
	2	74.6	2.0	81.8		2	75.0	2.2	98.3
HH-1	1	73.5	1.0	7.3	PL-1	1	73.3	1.2	1.8
	2	74.8	1.3	92.7		2	74.5	1.6	98.2
WG-1	1	73.4	1.5	10.9	PL-2	1	73.5	1.1	2.3
	2	75.0	2.1	89.1		2	74.6	1.6	97.7
HJD-1	1	73.4	1.2	6.7	GJL-1	1	73.4	1.5	1.7
	2	74.4	1.5	93.3		2	74.2	2.0	98.3
JH-1	1	73.5	1.2	6.4	GJL-2	1	73.4	1.3	1.8
	2	74.4	1.5	93.6		2	74.4	2.0	98.2

The fitting analysis of Al(2p) spectra reveals that Al in quartz from various geological backgrounds mainly existed as [AlO_6_]^0^ octahedra (Fig. 2 and Table 3). The [AlO_6_]^0^ octahedra in quartz are most likely attributed to its Al-containing impurity inclusions, suggesting the existence of many impurity fluids or solid inclusions in the quartz.^11,22,23^ To exclude the influence of impurity inclusions on the quartz thermometer, the relative proportions of [AlO_4_]^0^ tetrahedra (*k*) are calculated from the fitted peak areas of [AlO_4_]^0^ tetrahedra and [AlO_6_]^0^ octahedra (Table 3). The calculated *k* values range from 1.7% to 18.2%.

### 3.3 Oxygen isotope data

The *δ*^18^O values of quartz shown in Table 2 range from –2.8‰ to +18.3‰. Based on the quartz–water curve proposed by Matsuhisa *et al.* (1979), the equilibrium formation temperatures of quartz (*T*_E_) were measured by the oxygen isotope ratios (Table 2).^52–54^ The temperatures (*T*_E_) were calculated according to the following equation:

2 *δ*^18^O = 1000 ln *α*_QW_ = 3.34 × 10^6^*T*^−2^ − 3.31

for equilibrium isotope exchange with meteoric water (−10‰ = *T*_met_), oceanic water (*T*_oc_), and magmatic water (+8‰ = *T*_mag_), in which *α*_QW_ is the O-isotope fractionation factor between quartz and water, and *T* is expressed in K (Table 2).^55^ The results are retained as integers.

Based on published data, it is inferred that the mineral-forming fluids of quartz from two agate deposits were influenced by low-temperature hydrothermal fluid and meteoric water.^56^ Therefore, their formation temperatures are calculated to be 132 °C and 169 °C, respectively. However, the calculations of epithermal quartz vein SDWZ-1 for pure magmatic water led to isotope equilibration temperatures below 0 °C, so it seems to be unlikely (Table 2). Therefore, the meteoric water plays a dominant role during its formation, and an isotopic equilibrium temperature of 291 °C is selected as its formation temperature.^50^ The *δ*^18^O values of quartz from other gold and granodiorite deposits are much closer to those of quartz precipitated from magmatic fluids (Table 2). As a result, their formation temperature ranges are determined to be 222 °C to 417 °C and 535 °C to 560 °C, respectively. Consequently, the isotope compositions yield the calculated fractionation temperatures (*T*_E_) ranging from 132 °C and 560 °C.

### 3.4 Application of quartz thermometer

The results in Table 2 show that the total Al concentration in quartz (determined by ICP-OES) is greater than the concentration of [AlO_4_]^0^. This indicates the presence of Al-containing inclusions leading to the conclusion that the medium was saturated with respect to Al.^37^ The quartz is crystallized from the fluid under near-equilibrium conditions. Thus, the prerequisites for the application of quartz thermometers exist.^37^ The concentration of [AlO_4_]^0^ in quartz was derived by combining the data from ICP-OES and XPS. The calculated *k* is multiplied by the total Al concentration of quartz (*C*_Al total_) measured by ICP-OES to obtain the [AlO_4_]^0^ concentration in the quartz lattice (*C*_[AlO_4_]^0^_/ppm). The concentration of [AlO^4^]^0^ tetrahedra (*C*_[AlO_4_]^0^_/ppm) in the quartz lattice can be determined by the following equation:

3 *C*_[AlO_4_]^0^_ = *C*_Al total_ (ppm) × *k*

From agate to gold, and then to granodiorite deposits, the [AlO_4_]^0^ concentration in the quartz lattice (*C*_[AlO_4_]^0^_/ppm) increases from 33 ppm to 148 ppm. Combined with the research of Dennen *et al.* (1970),^35^ the formation temperature of quartz (*T*_Q_/°C) can be calculated from the following quartz temperature equation:

4 *T*_Q_(°C) = 3.6 × *C*_Al total_ (ppm) × *k* + 33.0

**Fig. 3:**
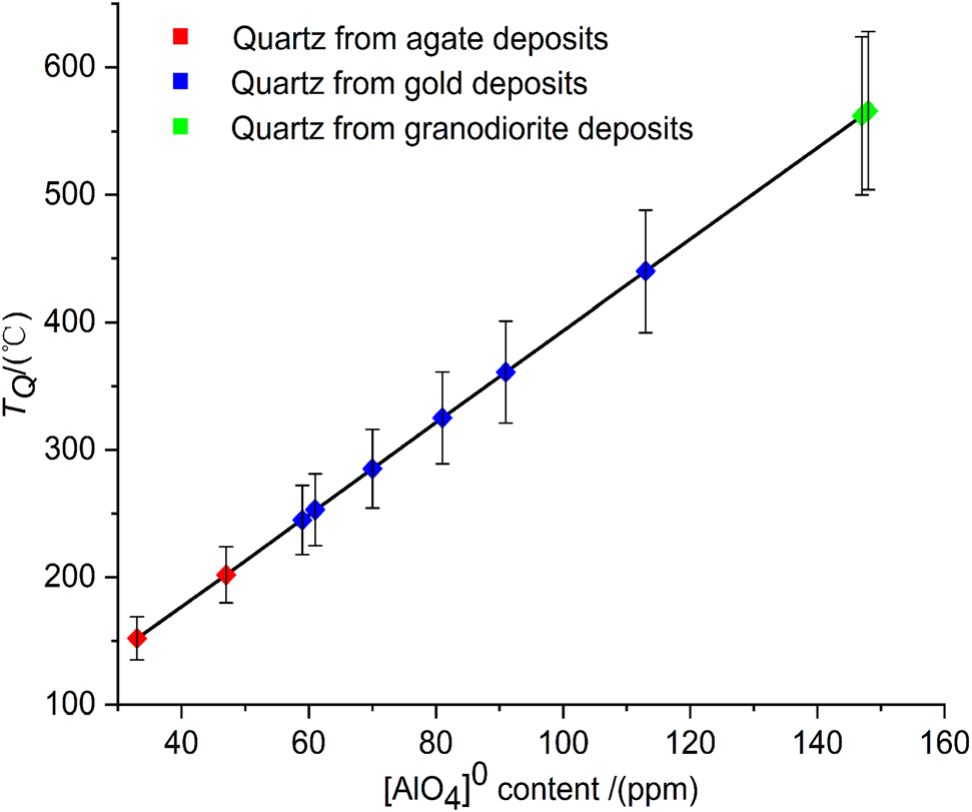
The formation temperatures of quartz calculated by the quartz thermometer with a relative error of less than ±11%.

The results are kept as integers. According to the error transfer equation, the relative error of the quartz thermometer is less than ±11% (Fig. 3). The calculated temperatures of quartz (*T*_Q_) are broadly distributed between 152 °C and 566 °C (Table 2). The formation temperatures of quartz from agate deposits are 152 and 202 °C, respectively. It is indicated that quartz pieces from these two agate deposits were formed under a low-temperature environment. Furthermore, quartz veins from gold deposits crystallize in the medium to high temperature range of 245 °C to 440 °C. The formation temperature of quartz in granodiorite is as high as 566 °C.

### 3.5 Reliability and advantages of quartz thermometer

**Fig. 4:**
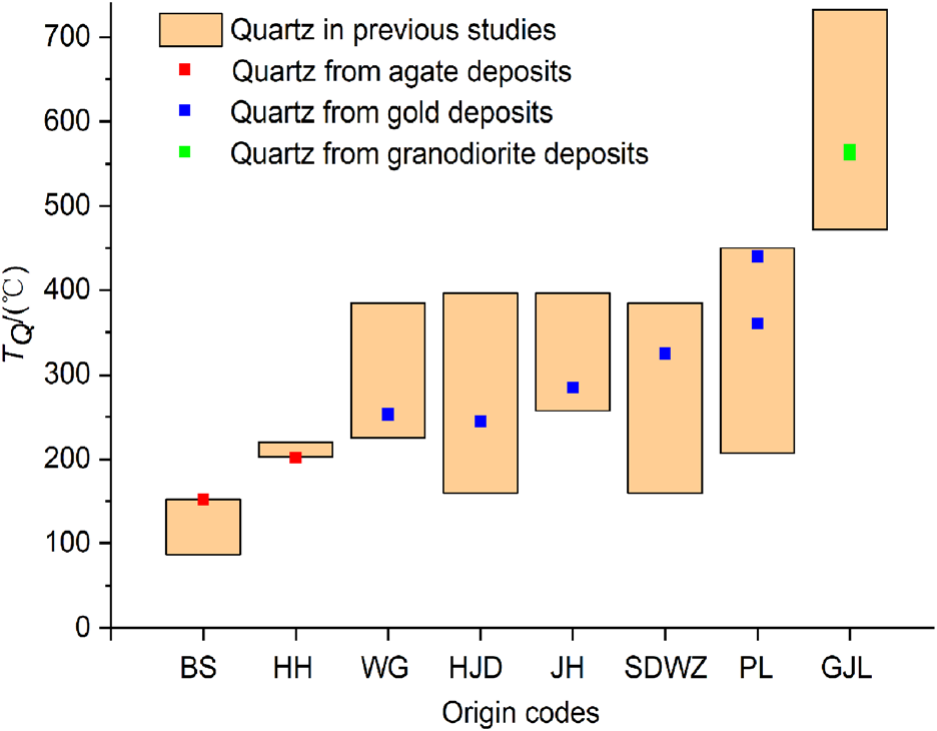
Comparison of the formation temperatures of quartz according to the quartz thermometer and published data, respectively.

The formation temperatures of quartz (*T*_Q_) calculated by the quartz thermometer are compared to the temperature ranges of quartz observed in previously published studies (Fig. 4). It is indicated that the *T*_Q_ values calculated by the quartz thermometer fall within the temperature ranges reported in previous studies. The temperature curves of quartz (*T*_Q_ and *T*_E_) calculated by the quartz thermometer and oxygen isotope data are shown in Fig. 4. The temperatures (*T*_Q_) calculated by the quartz thermometer are in good agreement with those (*T*_E_) obtained by oxygen isotope fractionation.^26^ In summary, the results demonstrate the reliability and applicability of the quartz thermometer for quartz of various origins and temperatures.

**Fig. 5:**
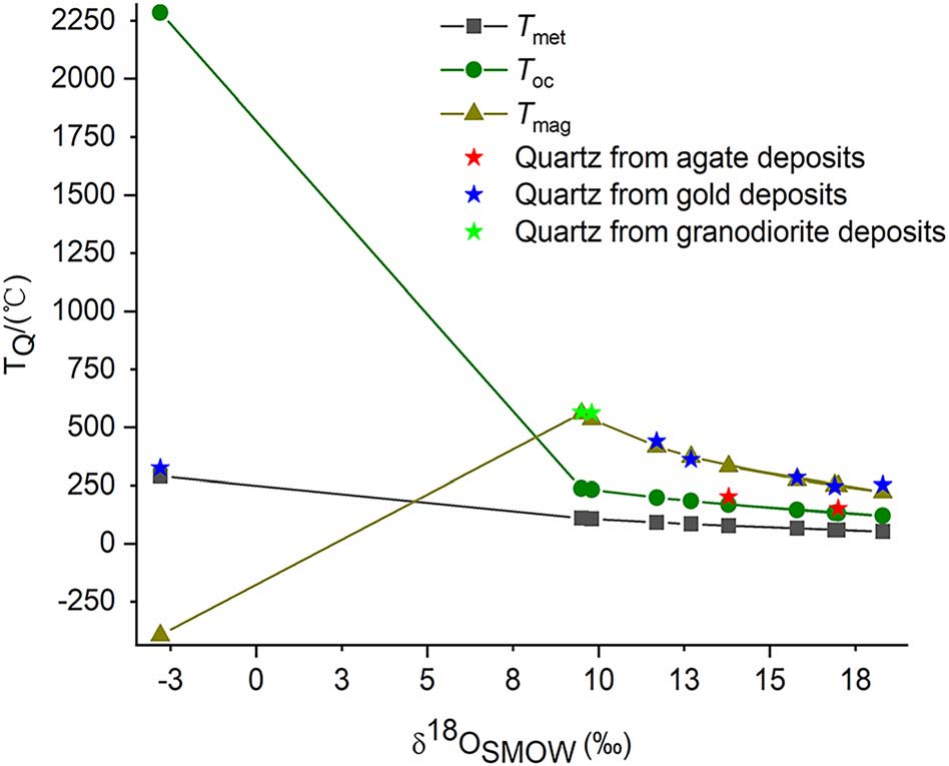
Comparison of the formation temperatures of quartz calculated from the quartz thermometer and oxygen isotope data.

The formation temperatures of quartz (*T*_Q_) in agate deposits determined by the quartz thermometer are plotted between the temperature curves in equilibrium with meteoric water and magmatic water (Fig. 5). This confirms the assumption that their mineral-forming fluids may be a mixture of meteoric and magmatic origin. According to the quartz thermometer, the formation temperature of epithermal quartz vein SDWZ-1 is closest to the temperature in equilibrium with meteoric water. It reveals that its mineral-forming fluid is dominated by meteoric water. The formation temperatures of quartz in other gold and granodiorite deposits calculated by the quartz thermometer are all located near the temperature curve in equilibrium with magmatic water. This provides further evidence of their magmatic origin. By comparing the relative positions of the two temperature curves (*T*_Q_ and *T*_E_), the composition of quartz mineral-forming fluids can be inferred. This provides valuable information for understanding the formation process of quartz and the origin of mineral-forming fluids.

The oxygen isotope analysis is commonly used to constrain the crystallization temperature of quartz in natural geological systems. When calculating the formation temperature of quartz (*T*_E_) from oxygen isotope data, it is crucial to deduce the composition of its mineral-forming fluids by analyzing its hydrogen isotope. The corresponding equilibrium temperature is then selected as its formation temperature. Alternatively, the process of fluid mixing, such as the mixing of meteoric water with late magmatic fluids, must be taken into account. However, the quartz thermometer can determine the formation temperature of quartz (*T*_Q_) without considering the composition of its mineral-forming fluids. This simplifies the process and reduces costs. Moreover, the quartz thermometer appears to be a more accurate and economical solution for quartz with mixed compositions of mineral-forming fluids.

## 4. Conclusions

In summary, the quartz thermometer combining ICP-OES and XPS demonstrates extensive applicability and serves as a powerful tool for investigating the formation temperature of quartz. It effectively eliminates the interference caused by microscopic inclusions in quartz and breaks the application limitations of the Al-in-quartz thermometer. Compared to other methods used for calculating quartz temperature, the quartz thermometer does not require complex pretreatment procedures and can provide a more accurate estimate of quartz formation temperature. Furthermore, by analyzing the relative positions of temperature profiles based on the quartz thermometer and oxygen isotope equilibrium, it is possible to infer the composition of quartz-forming fluids. This provides valuable information for studying the genesis of quartz.

## Author contributions

Mengmeng Shen: conceptualized; conducted experimental work and data analysis; drafted original manuscript. Mulin huang: edited the manuscript. Zhiyun Lu: conceptualized; edited the manuscript. Xuemei He: supervised the project, characterized properties of materials.

## Conflicts of interest

There are no conflicts to declare.

## Supplementary Material
